# Novel Dual Therapy: A Paradigm Shift in Anticoagulation in Patients of Atrial Fibrillation Undergoing Percutaneous Coronary Intervention

**DOI:** 10.1055/s-0040-1719081

**Published:** 2020-10-31

**Authors:** Akshyaya Pradhan, Monika Bhandari, Pravesh Vishwakarma, Rishi Sethi

**Affiliations:** 1 Department of Cardiology, King George Medical University, Lucknow, Uttar Pradesh, India

**Keywords:** atrial fibrillation, novel oral anticoagulants, high bleeding risk, randomized control trial, HAS-BLED score

## Abstract

Patients with atrial fibrillation (AF) on long-term oral anticoagulation (OAC) either have underlying coronary artery disease or suffer from acute coronary syndromes necessitating a percutaneous coronary intervention (PCI). In such a scenario, an amalgamation of antiplatelet and antithrombotic therapy (conventionally called as “triple therapy”) is obligatory for preventing coronary ischemia and stroke. But such ischemic benefits are accrued at the cost of increased bleeding. We also now know that bleeding events following PCI are related to increased mortality. Balancing the bleeding and ischemic risks is often a clinical dilemma. With the advent of novel oral anticoagulants (NOAC's) with preserved efficacy and attenuated bleeding rates, anticoagulation in AF is undergoing paradigm shift. The spotlight is now shifting from conventional triple therapy (vitamin-K antagonist + dual antiplatelet therapy [VKA + DAPT]) to novel dual therapy (NOAC + single antiplatelet therapy [SAPT]) in situation of anticoagulated AF patients undergoing PCI. Such a strategy aims to ameliorate the higher bleeding risk with conventional VKA's while retaining the ischemic benefits. In this review, we briefly discuss the need for combination therapy, trials of novel dual therapy, strategies for mitigating bleeding, the current guidelines, and the future perspectives in AF undergoing PCI with stent(s).

## Introduction

Atrial fibrillation (AF) is a common problem in cardiology practice with an increasing frequency day by day. It is the most common sustained arrhythmia seen in clinical practice. In 2010, approximately 20.9 million men and 12.6 million women were affected by AF. 1 The incidence, as well as prevalence of the disease was higher in developed countries. 2 The prevalence is estimated around to be 2% in individuals <65%, whereas in individuals, aged above 65 years, it is around 9%. 3 Individual with conditions, such as hypertension, heart failure, coronary artery disease (CAD), valvular heart disease, obesity, diabetes mellitus, or chronic kidney disease (CKD), are at high risk for AF along with advanced age. 1 2 AF is responsible for five-fold increased risk of strokes, two-fold increased risk of death and three-fold increased risk for heart failure. 4 5 6 7 8 The use of antithrombotic treatment along with control of risk factors have been found to reduce the risk of stroke substantially in patients with AF. 9

### The Need for Triple Therapy

Triple therapy (TT) refers to the concomitant use of an oral anticoagulant (OAC), such as warfarin and dual antiplatelet therapy (DAPT). 10 Such a therapy aims to simultaneously ameliorate risk of both coronary events (by antiplatelet component) and stroke (OAC component). Such therapy is most commonly indicated in patients with AF who develop acute coronary syndrome (ACS) or vice-versa and who undergo percutaneous coronary intervention (PCI) with stent insertion, which accounts for approximately 5 to 8% of all PCI patients. 10 Approximately, 30% of patients with AF have concomitant CAD and approximately 15% of them will PCI during their lifetime. 9 Optimal antithrombotic treatment for patients with AF undergoing PCI is one of the hot topics in cardiology.

Guideline directed TT is known to improve clinical outcomes in patients with AF or CAD; however, it is associated with increased risk of bleeding. 9 The rationale for prescribing TT in this scenario is two-fold, DAPT has been shown to be superior to an OAC for reducing the risk of stent thrombosis. 11 However, DAPT is inferior to an OAC for preventing thrombotic events in patients with AF. 11 Thus, theoretically TT seems to be necessary in these patients and is empirically indicated in AF patients undergoing PCI. However, no randomized control trials have tested the efficacy of TT but it is well known to increase the risk of both fatal and nonfatal bleeding.

### Bleeding Risk with Combination Therapy

**Fig. 1:**
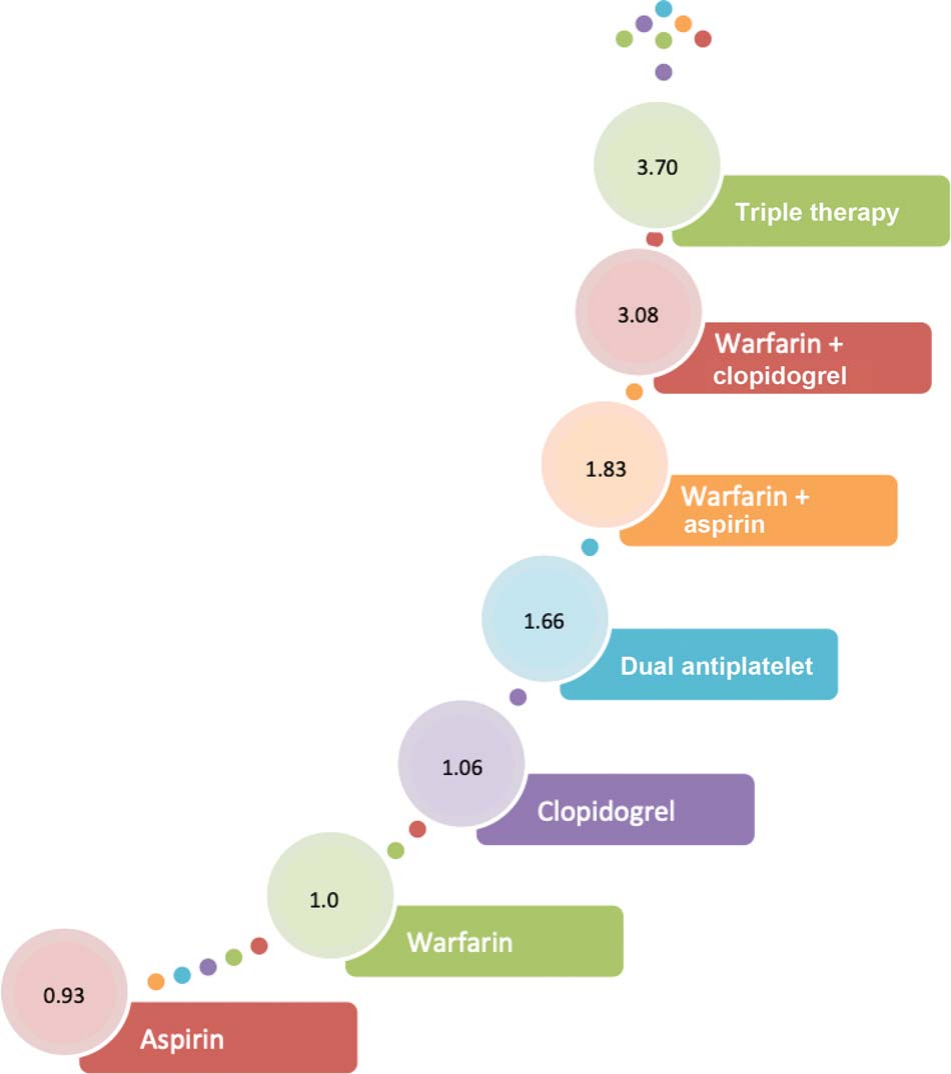
Hierarchical hazard of bleeding with anticoagulant and antiplatelet agents with warfarin as standard/reference. The numbers in the circle against each regimen represents the Hazard ratio for bleeding. 14 Dual antiplatelet refers to aspirin + clopidogrel; triple therapy refers to dual antiplatelet + vitamin-K antagonist.

Although, TT reduces the risk of stent thrombosis, it is evident from observational studies that it poses great risk of serious bleeding. The rate of major bleeding was increased from 1.3 to 1.9% by concomitant use of antiplatelet drugs in study by Shireman et al. 12 Similarly, another study by Johnson et al showed that higher number of patients in the combination-therapy group had anticoagulation-related hemorrhagic events compared with patients receiving monotherapy (4.2 vs. 2.0%, *p* < 0.001). 13 Hansen et al studied the risk of bleeding with single, dual, and TT. 14 They found that the risk of bleeding is doubled when aspirin is combined with OAC as compared with warfarin monotherapy. Also, DAPT caused more bleeding and ischemic strokes then warfarin monotherapy. Clearly warfarin monotherapy was more effective in preventing ischemic strokes in AF with less bleeding risk. The hierarchical risk of bleeding with antiplatelet and anticoagulant agents as monotherapy and in combination is depicted in Fig. 1 .

### Post-PCI Bleeding Is Not Benign

**Fig. 2:**
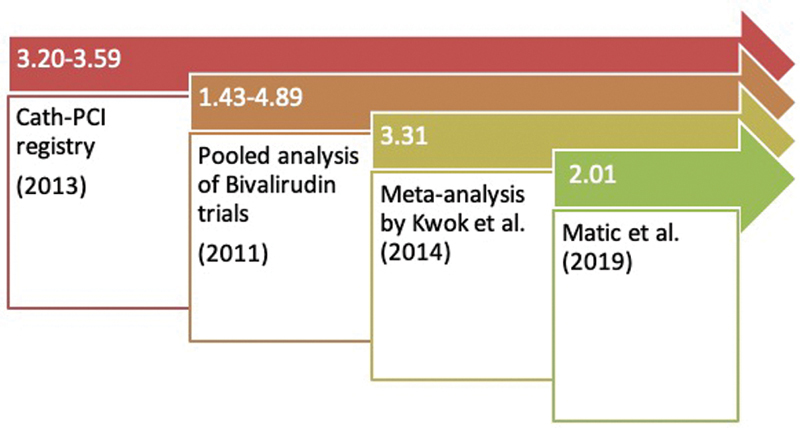
Post-PCI bleeding increases mortality as seen by the increased hazard ratio's across various studies. 15 16 17 18

Bleeding is a common complication following PCI and doubles up as a major cause morbidity amongst them. In the data from, Cath PCI registry of 3,386,688 which analyzed PCI patients from 2004 to 2011, there were 57,246 (1.7%) bleeding events. The risk of mortality related to major bleeding was 12.1% in the PCI cohort. 15 Mehran et al studied 17,034 patients who underwent PCI from three major trials of bivalirudin vs heparin plus GPIIB/IIIA inhibitors. Non-CABG (coronary artery bypass grafting)–related major bleeding occurred in 267 patients (1.6%) within 30 days post-PCI and 2.9% patients died within 1 year. 16 In a meta-analysis and systemic review of PCI studies, PCI-related major bleeding was responsible for three-fold increase in mortality. 17 Also, primary PCI associated access and nonaccess site bleeding is major concern of mortality and morbidity. 18 As seen in Fig. 2 , the hazard ratio for mortality due to bleeding post PCI varies approximately from 2.00 to 4.00 in various studies.

### Connection between Post-PCI Bleeding and Mortality

**Fig. 3:**
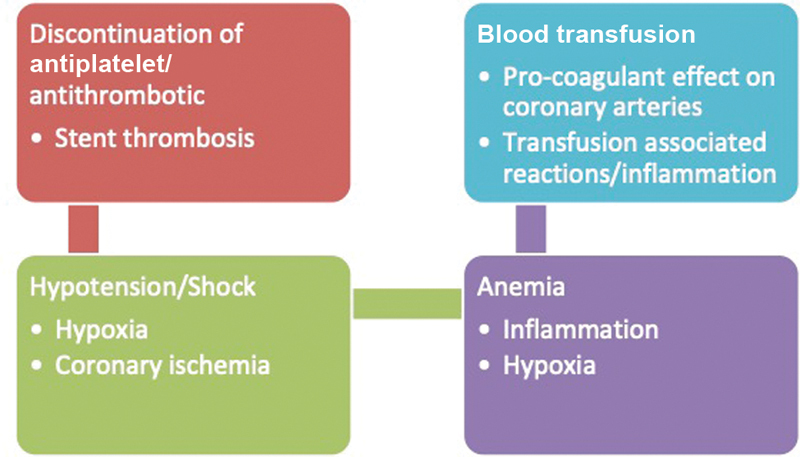
Plausible mechanisms contributing to mortality after bleeding events following PCI. PCI, percutaneous coronary intervention.

There are multiple plausible mechanisms linking bleeding events with mortality ( Fig. 3 ).Bleeding leads causes hypotension and anemia which causes hypoxemia leading to myocardial ischemia. Severe blood loss will require blood transfusion. Transfusion of blood may trigger inflammatory cascade which will lead to vasoconstriction and ischemia. In addition, discontinuation of DAPT will predispose to stent thrombosis. All of these together ultimately causes an increase in morbidity and mortality. In addition, if patient has intracranial bleeding, patient may have mass effect which can lead to patient's death. Thus, bleeding in the setting of PCI is not at all benign, and it mitigates the advantages of revascularization, especially in the setting of ACS. Hence, every effort should be made to decrease the risk of bleeding in PCI patients which is especially important where we need anticoagulant therapy with DAPT. 19 20

### Alternative to Triple Therapy: OAC Plus Single Antiplatelet Therapy

To reduce bleeding and its serious consequences in patients of AF who require PCI, WOEST trial studied effects of dual therapy (DT; clopidogrel with warfarin) and ISAR Triple evaluated an abridged version of TT (6 weeks). WOEST trial was an open label randomized control trial (RCT) of 563 patients who had long-term indication for anticoagulation with severe CAD requiring PCI. 21 Patients were randomized to receive either clopidogrel alone with warfarin (DT) or clopidogrel along with aspirin and warfarin (TT). After 1-year follow-up, 44.4% patients in TT group and 19.1% in double therapy group had bleeding as adjudged by thrombolysis in myocardial infarction (TIMI) scale (hazard ratio [HR] = 0.36, 95% confidence interval [CI]: 0.26–0.50, *p* < 0.0001).The incidence of recurrent bleeding and bleeding requiring transfusion was also less in DT arm compared with TT arm. At the same time, the secondary-efficacy end point of major adverse cardiovascular events including stent thrombosis and target vessel revascularization was attenuated in the DT arm (HR = 0.39, 95% CI: 0.16–0.93, *p* = 0.025). However, the small sample size allowed adequate power to detect differences in bleeding events but not for secondary ischemic end points. Moreover, the improvement in safety of DT was primary driven by TIMI minor/minimal bleeding without any significant differences in major bleeding. The open label design of the study was also a concern which may have led to error of outcomes reporting both by physician and patients' alike. 22 Despite the criticisms, the study exposed research gaps in the area of AF with PCI and raised serious concerns regarding the practice of 1-year TT following PCI in AF patients.

ISAR-Triple study tested a different strategy of tailoring the duration of TT to decrease bleeding. In this open label RCT, 614 patients on oral anticoagulation (OAC) who underwent PCI were randomized to two different regimens of clopidogrel of 6 weeks and 6 months. 22 Concomitant administration of aspirin and vitamin-K antagonist (VKA) was unchanged in both arms. At 9 months, the primary end point composite of death, myocardial infarction (MI), definite stent thrombosis, stroke, or TIMI major bleeding was not different between the two arms. Small sample size and an open-label design again were major limitations.

Although observational studies had shown reduced bleeding with warfarin-based DT over TT, the two randomized studies failed to establish a definitive place for DT in the scenario of AF with PCI or ACS.

### Dual Therapy with NOAC: A Paradigm Shift

Warfarin has remained the cornerstone stone for thromboprophylaxis in patients with AF for decades. However, its use is cumbersome because it needs monitoring (international normalized ratio [INR]) which is quite difficult sometimes. This is especially true in developing countries where the majority of patients reside in rural areas where facilities of INR monitoring is not available which can lead to under or overdosing with dire consequences. The evolution of novel OAC agents (NOACS) which include direct thrombin inhibitor dabigatran and factor X-A inhibitors, like apixaban, rivaroxaban, and edoxaban, have changed the treatment of patients with AF. NOACs have multiple pharmacologic edges over warfarin, which include rapid onset/offset of action, few drug interactions, and predictable pharmacokinetics. 10 In addition, warfarin has a narrow therapeutic range which is influenced by factors, such as diet, and obviously requires INR monitoring. The results of various trials have demonstrated better safety and at least similar efficacy to warfarin in preventing ischemic strokes. A meta-analysis of 12 studies of NOACS involving 77,011 patients demonstrated that with NOACS, there was a significant reduction in incidence of ischemic stroke and systemic emboli (odds ratio [OR] = 0.85, 95% CI: 0.75–0.98). 23 There was 52% reduction in intracranial hemorrhage (ICH; OR = 0.48, 95% CI: 0.40–0.57) and 14%reduction in mortality (OR = 0.86, 95% CI: 0.82–0.91). While switching to warfarin caused increase in both stroke (OR = 2.60, 95% CI: 1.61–4.18) and bleeding events (OR = 2.19, 95% CI: 1.42–3.36). Another meta-analysis of 18 RCTS with 78,796 patients of NOACS comparing warfarin in AF patients showed reduction in ischemic strokes with NOACS with highest *P* score for dabigatran 150 mg followed by apixaban 5 mg. 24 The risk of ICH and bleeding events were lower for all NOACS as compared with warfarin with highest *P* score for edoxaban 30 mg followed by apixaban 5 mg.

**Fig. 4:**
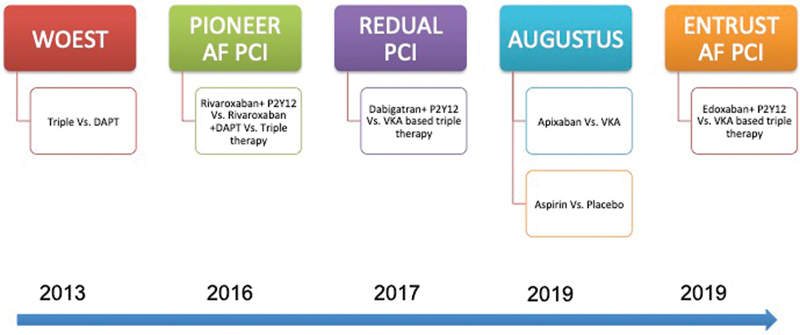
Timeline of pivotal trials comparing dual therapy vis-à-vis triple therapy following AF undergoing PCI. AF, atrial fibrillation; DAPT, dual antiplatelet therapy; PCI, percutaneous coronary intervention; VKA, vitamin-K antagonist.

So, clearly, NOAC's stand ahead of warfarin in terms of both efficacy and safety. Hence, the use of an NOAC in place of VKA for combination therapy (dual or triple) has potential advantages with the aim of targeting the main pitfall of warfarin based TT, that is, bleeding complication. Four large RCT's described below have now tested this novel DT (NOAC + P2Y12 inhibitor) against the conventional DT/TT based on a warfarin ( Fig. 4 ; Table 1 ).

**Table 1:** Salient features of major RCT's comparing dual therapy with conventional triple therapy

Name of study	Year	Comparators	Patients	Duration (mo)	Primary end point
WOEST 56	2013	VKA + C VKA + DAPT	563	12	Total number of TIMI bleeding events
ISAR TRIPLE 22	2015	VKA + DAPT: 6 wk VKA + DAPT: 42 wk	614	9	Composite of death, MI, definite stent thrombosis, stroke and TIMI major bleeding
PIONEER AF 10	2016	R5 + C R2.5 + C VKA + DAPT	1,415	12	A composite of major bleeding or minor bleeding according to the TIMI or bleeding requiring medical attention
RE-DUAL PCI 57	2017	D110 + C D150 + C VKA + DAPT	2,725	24	A composite of major or clinically relevant nonmajor bleeding event according to ISTH
AUGUSTUS PCI 26	2019	VKA + C NOAC + C VKA + DAPT	4,614	6	A composite of major or clinically relevant nonmajor bleeding event according to ISTH
ENTRUST AF 27	2019	NOAC + C VKA + DAPT	1,506	12	Major or clinically relevant nonmajor bleeding event according to ISTH

Abbreviations: C, clopidogrel; D, dabigatran; DAPT, dual antiplatelet therapy; ISTH, International society of Thrombosis and Hemostasis; NOAC, novel oral anticoagulant; R, rivaroxaban; RCT, randomized controlled trial; TIMI, thrombolysis in myocardial infarction; VKA, vitamin-K antagonist.

## PIONEER AF PCI

It was the first RCT which studied three strategies of antiplatelets and anticoagulant therapy in patients of AF undergoing PCI. 25 A total of 2,124 patients were included in the study and followed up to 12 months. Patients were randomized to either rivaroxaban plus P2Y12 inhibitor (novel DT), rivaroxaban plus DAPT (novel TT), or to warfarin plus DAPT (conventional TT) for 12 months. The doses of rivaroxaban in DT arm was 15 mg once daily and in 2.5 mg twice daily in TT arm. Three prespecified durations of 1 month, 6, and 12 months for DAPT were also tested simultaneously in the TT arms. The incidence of primary end point, that is, clinically significant bleeding, occurred in 16.8% of patients in group 1 versus 18.0% group 2 versus 26.7% in group 3 (HR = 0.59, *p* < 0.001 for group 1 vs. group 3; HR = 0.63, *p* < 0.001 for group 2 vs. Group 3). Major bleeding was significantly higher in group 3 (2.1 vs. 1.9 vs. 3.3%). The reduced bleeding with rivaroxaban was consistent across all subgroups also.

However, major adverse cardiovascular events (MACE) and stent thrombosis were not different in all the three groups (MACE: 6.5 vs. 5.6 vs. 6.0%, *p* = 0.75; ST: 0.8 vs. 0.9 vs. 0.7%, *p* = 0.79). Stent thrombosis was 1.9% in group 2 patients who received DAPT for 1 month compared with 1.7% who got DAPT for 6 months (was nil for DAPT of 1 year). So, the trial convincingly demonstrated that rivaroxaban based DT in AF patients who undergo PCI significantly reduce bleeding and rivaroxaban with single antiplatelet drug is equally effective to DAPT in reducing stent thrombosis. 10

## RE-DUAL PCI

Following the successful demonstration of feasibility of the PIONEER AF PCI, RE-DUAL PCI was a study intended to further explore the DT. It was a randomized, parallel, stratified trial of 2,725 patients with AF who received percutaneous coronary revascularization with a stent. 27 The trial pitted two doses of DT with dabigatran 110 mg twice daily ( *n* = 981) and 150 mg twice daily with P2Y12 inhibitor versus conventional TT with warfarin with P2Y12 inhibitor and aspirin ( *n* = 981). The duration of aspirin was 1 month after a bare metal stent/drug eluting stent and 3 months after a DES.

The primary end point (major or clinically relevant nonmajor bleeding events) occurred in 15.4% of patients who received dabigatran 110 mg twice day compared with 26.9% in the TT group ( *p* for noninferiority <0.001 and *p* for superiority <0.001). Similarly, the rates of both major and clinically relevant nonmajor bleeding were reduced with 150-mg dose of dabigatran as compared with warfarin based TT (20 vs. 27.5%, respectively [ *p* for noninferiority <0.001]). TIMI major bleeding was also lower in the DT versus TT group. The secondary efficacy outcome in the study included incidence of death, MI, stroke, systemic embolism, or unplanned revascularization. These efficacy outcomes were not different between dual and TT cohorts (13.7 vs. 13.4%; *p* for noninferiority = 0.005). The incidence of stent thrombosis was similar in dabigatran 150-mg dose as TT (0.9%), while it was insignificantly higher with 110-mg dose of dabigatran (1.5 vs. 0.8% [ *p* = 0.15]). In subgroup analysis, it was found that patients who received ticagrelor had higher bleeding events as compared with clopidogrel. This result is consistent with the data from ticagrelor studies where the drug has shown to have higher bleeding not related to CABG.

Thus, among patients with AF undergoing PCI, novel DT with dabigatran was significantly able to reduce bleeding events albeit at the cost of insignificant rise in stent thrombosis with lower dabigatran doses. With the initial success of novel DT with both dabigatran and rivaroxaban, the stage was now set for the remaining two NOAC's to enter the fray.

## AUGUSTUS PCI

It was the third RCT in the series which additionally explored the role of aspirin in the scenario of post-PCI/ACS with AF. It was a 2 × 2 factorial designed trial in which patients with AF developed ACS or had PCI and were randomized in a 1:1 fashion to either apixaban 5-mg BID ( *n* = 2,306) or VKA with an INR goal of 2 to 3 ( *n* = 2,308) with either aspirin 81-mg daily ( *n* = 2,307) or matching placebo ( *n* = 2,307) for at least 6 months. 26 All patients received a P2Y12 inhibitor. The patients were followed for up to 6 months. The International Society on Thrombosis and Hemostasis (ISTH) major or clinically relevant nonmajor bleeding for apixaban versus VKA was 10.5 versus 14.7%, ( *p* < 0.0001).While, ISTH major or clinically relevant nonmajor bleeding for aspirin versus placebo, was 16.1 versus 9.0%, *p* < 0.0001.The secondary outcome of death or hospitalization for apixaban versus VKA was 23.5 versus 27.4%, *p* = 0.002, while for aspirin versus placebo it was 26.2 versus 24.7%, *p* > 0.05. The incidence of stent thrombosis in two arm were not significantly different (0.74% for apixaban vs. 0.97% for VKA). Thus, in comparison to VKA, apixaban caused significant reduction in ISTH major and clinically significant bleeding events in ACS patients treated either medically (HR = 0.44, 95% CI: 0.28–0.68) or with PCI (HR = 0.68, 95% CI: 0.58–0.89). Addition of aspirin was associated with increased bleeding events in both the arms without any major difference in efficacy.

## ENTRUST AF PCI

**Fig. 5:**
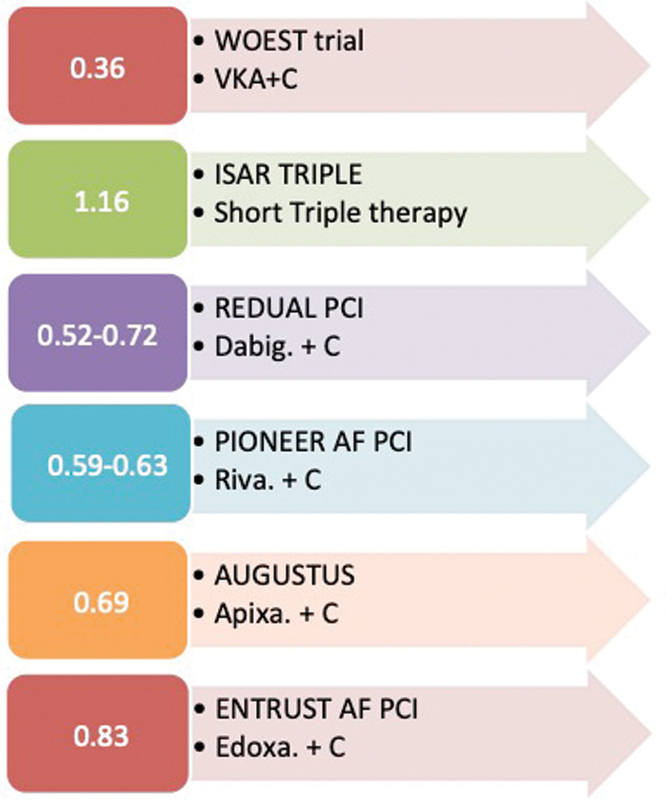
Diminished hazard of bleeding with dual therapy (OAC + SAPT) with triple therapy (VKA + DAPT) as a standard /reference. C, clopidogrel; DAPT, dual antiplatelet therapy; SAPT, single antiplatelet therapy; VKA, vitamin-K antagonist.

With this study, we have RCT's on all the available NOAC currently approved for AF. This trial compared edoxaban and clopidogrel combination with VKA plus DAPT among patients with AF who recently underwent PCI. 27 Patients with AF and recent PCI were randomized to edoxaban 60-mg daily plus clopidogrel 75-mg daily for 12 months ( *n* = 751) versus a VKA and clopidogrel 75-mg daily for 12 months plus aspirin (100-mg once daily, for 1–12 months; *n* = 755). Lower dose of edoxaban, 30-mg daily, was used in patients with creatinine clearance <15 to 50 mL/min, weight <60 kg, or concurrent use of specific potent P-glycoprotein inhibitors. The rates of major or clinically relevant nonmajor bleeding at 12 months, was 17% in edoxaban group compared with 20% in the VKA group (HR = 0.83, 95% CI: 0.65–1.05, *p* for noninferiority = 0.001, *p* for superiority = 0.12). While the rate of cardiovascular death, myocardial infarction, stroke, systemic embolism, or definite stent thrombosis was 7% with edoxaban versus 6% with VKA ( *p* = not significant). Although this trial did show less bleeding with DT using NOACS as compared with VKA, but it did not meet the superiority criteria. The hazards of bleeding events for DT versus TT in various studies are depicted in Fig. 5 .

### Meta-analysis of Dual Therapy

There are a few meta-analyses of the randomized trials which compared DT with TT in AF patients undergoing PCI. Golwala et al did a meta-analysis of patients from WOEST, ISAR TRIPLE, PIONEER AF PCI, and RE-DUAL PCI trials. 28 A total of 5,317 patients from these four RCT's were included of whom 3,039 (57%) received DT. 28 There was a 47% reduction in the incidence of TIMI major and minor bleeding in DT regimen (4.3 vs. 9.0%, HR = 0.53, 95% CI: 0.36–0.85) without any major difference in the incidence of major adverse cardiovascular events (10.4 vs. 10%, HR = 0.85, 95% CI: 048–1.29). Notably, ENTRUST AF and AUGUSTUS PCI were not published till date and hence not included in this meta-analysis.

Further credence to DT was provided by a network meta-analysis by Lopes et al. They included 10,026 patients from WOEST, RE-DUAL PCI, PIONEER AF PCI, and PIONEER AF PCI. 29 TIMI major bleeding was the primary bleeding outcome while MACE was the principal efficacy outcome studied. Compared with TT, the odds of bleeding were lowest for NOAC based DT (HR = 0.49; 95% CI: 0.30–0.82) followed by VKA-based DT (HR = 0.58, 95% CI: 0.31–1.0) and NOAC based TT (HR = 0.70, 95% CI: 0.38–1.23), respectively. With respect to efficacy, the odds of MACE with NOAC–based DT, VKA-based DT and NOAC-based TT were 1.02, 0.96, and 0.94, respectively. Hence, NOAC-based DT or novel DT had the maximum safety record with preserved efficacy. Edoxaban was missing in action in the study as ENTRUST AF PCI had not yet seen light of the day.

More recently, in 2019, a meta-analysis published in in European heart journal included 10,234 patients from all the four pivotal RCT's of NOAC-based DT versus TT with in AF patients undergoing PCI also mirrored similar results. 30 They found that ISTH major and clinically relevant bleeding was significantly lower in DT arm vis-à-vis TT (risk ratio [RR] = 0.66, 95% CI: 0.56–0.78; *p* < 0.0001; *I* ^2^ = 69%). The benefit was primarily driven by significant reduction in intracranial hemorrhages (RR = 0.33, 95% CI: 0.17–0.65; *p* = 0.001). All-cause death, cardiovascular death, and MACE rates were not different between the novel DT and TT arms.

Lastly, another meta-analysis of latest trials of NOAC's (PIONEER-AF, RE-DUAL PCI, and AUGUSTUS trial) which included 9,463 patients also emphasized that NOAC-based regimens (DT or TT) were associated with significant reduction in bleeding events as compared with VKA-based therapeutic regimens. 31 With regard to ischemic events including stroke and death, the results were no different between DT and TT. However, there was a nonsignificant increase in rates of MI with DT, while a significant increase in stent thrombosis was observed with DT. This was primarily driven by lower dabigatran doses (110 mg). Interestingly, the results were unrelated with type of OAC used in DT (VKA or NOAC). The authors advocate the use of initial NOACS-based TT in AF patients undergoing PCI to curtail the incidence of stent thrombosis with DT. Attention was also drawn toward the benefits of using the full dose NOACS to achieve the maximum bleeding benefit and least ischemic events.

So, it can be reasonably concluded from these major meta-analysis that NOACS based DT is as efficacious as VKA plus P2Y12 therapy in terms of preventing both ischemic events with added advantage of safety in terms of both major and minor bleeding events.

### A Word of Caution

However, there was a signal of increased stent thrombosis and MI in the DT group in two of the above mentioned metanalyses. 30 31 Interestingly, the individual RCT's did report any such signal owing to lesser frequency of ischemic events as compared with bleeding events (approximately 10-fold fewer).

A point of interest is that none of the RCT's discussed above were powered for ischemic endpoints and were primarily designed to test the safety of aspect of DT ( Table 1 ). This signal of increased cardiac ischemic events was mainly restricted to lower doses of dabigatran (110 mg).

One has to remember that NOAC's are still not approved for use in pregnancy, severe mitral stenosis and in patient with prosthetic metallic valves. 11 Therefore, is such situations a conventional dual or TT with warfarin would still be recommended.

### Concomitant Strategies for Mitigating Bleeding

Bleeding remains a major impediment for combination of antiplatelet and antithrombotic therapy. As evident from the prior discussion, such bleeding after PCI increases the odds of dying and prolonged hospital stay with attendant financial implications.

Optimizing the time in therapeutic range (TTR) for patients put on VKA-based therapy can be useful in reducing bleeding episodes, as well as major adverse cardiac and cerebrovascular events (MACCE). In a registry of 963 AF patients undergoing PCI, 470 patients were divided into three TTR tertiles (T1, T2, and T3). It was found that patients with highest TTR (T3) had lowest bleeding events with progressive decline from T1 to T3. Also, there was a trend toward insignificant decrease in MACCE from T1 to T3. 32 Hence, the role of optimal control in quality of anticoagulation for diminishing bleeding cannot be over emphasized.

Apart from use of NOAC's in place of VKAs, five other key maneuvers that help to limit the bleeding include use of radial access, keeping doses of aspirin low, use of proton pump inhibitor (PPI), deescalation of P2Y12 therapy, and shorter duration of DAPT. 33

Use of radial access has brought a paradigm shift in access site complications cardiac catheterization laboratory. Apart from patient comfort and early ambulation, a significant and drastic reduction of access site bleeding has been the most gratifying revelation of radial access. MATRIX study is one the most contemporary and largest RCT demonstrating reduction in not only bleeding but also death, MI, and stroke at 30 days with radial access. 34 Of note there was 28% reduction in all-cause mortality and 33% reduction in BARC three or five major bleeding.

Aspirin has been used in doses from 50 mg to 325 mg for antiplatelet effects. However, the beauty is that lower doses (<100 mg) of drug has been consistently found to be as useful as the higher doses albeit with lesser bleeding. 35 36 Moreover, higher doses of aspirin interfere with antiplatelet action of ticagrelor. 37 Hence, use of lower doses aspirin in prudent when combination therapy with anticoagulants is contemplated.

Concomitant use of PPI is indicated in patient's on DAPT when they have prior gastrointestinal bleeding or they have high risk features for it. 38 39 The high risk features include advanced age, use of OACs, coprescription of nonsteroidal anti-inflammatory drugs (NSAIDs), and steroids. Hence, whenever contemplating DT or TT in a patient, it is advisable to use a PPI. The COGENT trial has already laid to rest the concerns about interaction between PPI and clopidogrel. 40

The type of P2Y12 used in the dual and triple regimen also is crucial. Although, more potent the newer agents, prasugrel and ticagrelor are more prone for bleeding including gastrointestinal (GI) bleeds. Even in the four major RCT's discussed prori, clopidogrel was the major antiplatelet used and the newer agent was used only in a minority. A single-center cohort study of 377 patients on TT, prasugrel-based regimens exhibited more TIMI defined major and minor bleeding compared with clopidogrel one's (28.6 vs. 6.7%; HR = 4.6, *p* = 0.03). 41 The TRANSLATE-ACS study compared clopidogrel-based TT with prasugrel-based TT. 42 Prasugrel-based therapy again increased the bleeding events defined by Bleeding Academic Research Consortium (BARC) scale. However, this was driven mostly by an increased risk of bleeding events that were patient-reported only and did not require rehospitalization. Hence, it would be more prudent to use clopidogrel when combining P2Y12 with anticoagulants to minimize the bleeding risk.

However, sometimes the use of Potent P2Y12 may be unavoidable because of high angiographic or procedural reasons such as complex PCI, use of multiple stents, left main interventions, bifurcation PCI, chronic total occlusion, long stents, and use of bioabsorbable scaffold. 43 In such a case, a “de-escalation” strategy for P2Y12 inhibitors can be applied. This strategy entails the switching of prasugrel or ticagrelor to clopidogrel after initial high-risk period after PCI is over to reduce the bleeding. Such a deescalation may be guided by platelet function (TROPICAL-ACS model) or unguided (TOPICS model). In TROPICAL-ACS trial, platelet function testing guided switch from prasugrel to clopidogrel at 14 days after PCI for ACS was shown to feasible and safe while reducing BARC bleeding. 44 In TOPICS study, an unguided deescalation 1 month after PCI was also shown to reduce bleeding events while ischemic were not increased. 45

Irrespective of the P2Y12 used, data are emerging on use of shorter duration of DAPT following PCI for ACS instead of conventional 12-month duration mandated by guidelines. REDUCE and DAPT STEMI trials have demonstrated the safety and feasibility of 3- and 6-month DAPT following PCI for ACS. 46 The small sample size and the selective population studies preclude generalization of results. More recently, STOP DAPT-2 and SMART CHOICE reaffirmed the safety of 1- and 6-month DAPT regimen following PCI. 47 48 Both the studies included an all-comer population, used newer generation stents, contemporary PCI techniques, and had a fair sample size. Although, patients with need for anticoagulation were excluded from STOP DAPT-2 study, a shorter DAPT remains one of the viable options for curtailing bleeding risk with combination therapy.

**Fig. 6:**
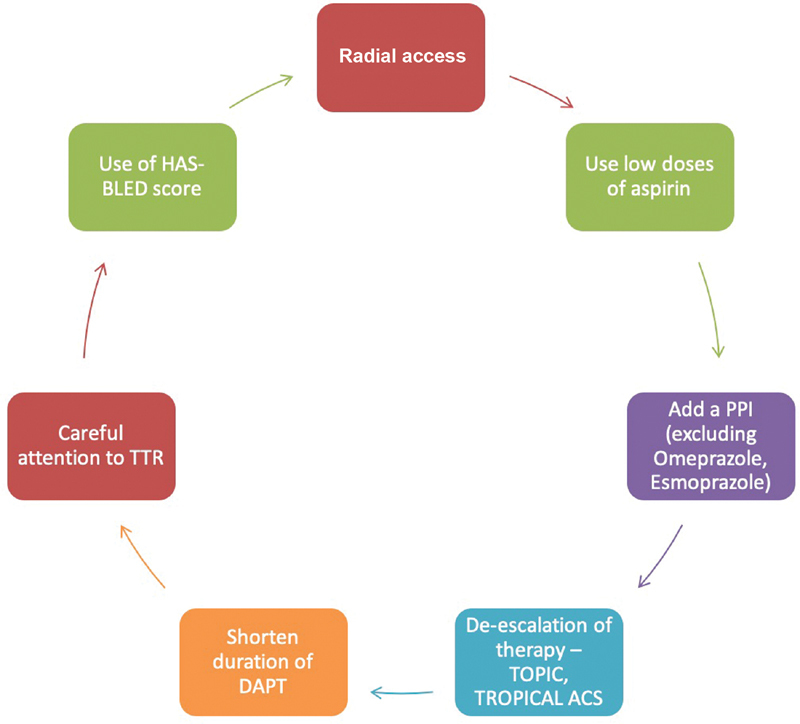
Steps for mitigating the bleeding risk in patients on a combination of antiplatelet and antithrombotic therapy. ACS, acute coronary syndrome; DAPT, dual antiplatelet therapy; PPI, proton pump inhibitor; TTR, time in therapeutic range.

Fig. 6 summarizes the additional maneuvers for mitigating bleeding with combination therapy.

### Use of Bleeding Prediction Scores

**Fig. 7:**
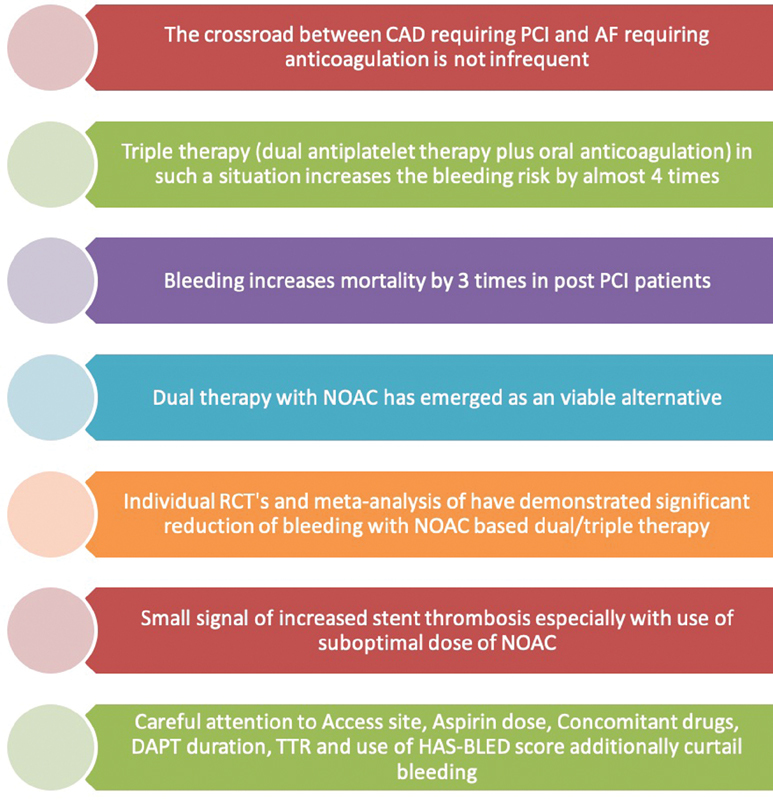
Key messages regrading use of dual therapy in the setting of PCI in AF patients on chronic Oral Anti Coagulation. AF, atrial fibrillation; CAD, coronary artery disease; NOAC, novel oral anticoagulant; PCI, percutaneous coronary intervention; TTR, time in therapeutic range; RCT, randomized controlled trial.

A lot of bleeding prediction scores are in vogue and also advocated by guidelines for clinical decision making ( Fig. 7 ). One needs to understand the precise subset where the individual score should be used.

The PRECISE-DAPT score predicts the rate of in hospital bleeding with the use of DAPT therapy. A score of <25 predicts the safety of prolonged DAPT. 33 Hence, the score can be utilized to decide the intensity of DAPT component of TT. On the other hand, HAS-BLED score predicts the bleeding on patient who are anticoagulated for AF. A score of >3 indicated high-bleeding risk and warrants preventive therapy. 49 Hence, patients with HAS-BLED score of >3 are definite candidate for NOAC, although this has not been prospectively tested. However, going by the previously described data of 50% reduction in ICH by NOAC's, the statement becomes imperative.

A systematic review has analyzed the current tools for thromboembolic and bleeding risk prediction in patients with AF. 50 The five bleeding risk scores evaluated were as follows: bleeding risk index (BRI), age, biomarkers and clinical history (ABC) bleeding risk score, HAS-BLED, ATRIA, and HEMORR _2_ HAGES among 38 studies of AF. The authors concluded that HAS-BLED score has the highest predictive value for major bleeding among patients on VKA's as assessed by c-statistic.

It needs to be emphasized here that bleeding risk is a dynamic variable and can evolve over time. The change in HAS-BLED score in a patient during follow-up can be more predictive of bleeding than baseline score itself. 51 In a nationwide cohort of 19,600 patient with an AF on VKA with low HAS-BLED score (<2 at baseline), during follow-up change is HAS-BLED >1 was seen in 76.6% in patients who experienced major bleeding. The proportion of subjects with change in HAS-BLED >1 (delta HAS-BLED score) was significant lower in group sans any bleeding events. The number of modifiable risk factors at baseline also predictive of bleeding events though not achieving statistical significance. In a similar manner, the dynamic change in CHA2DS2VaSc score during follow-up was also more predictive of ischemic risk vis-à-vis the baseline CHA2DS2VASc score. 52

Both the studies exemplify the role of evaluation of continued assessment of bleeding and ischemic risk at follow-up visits. Hence, a high HAS-BLED score cannot be an excuse for omitting OAC from prescription and only indicates the need for careful assessment of choice and dose of OAC while devoting careful attention to presence of modifiable risk factors for bleeding. It should also flag the caregivers for early review and meticulous follow-up in the high-risk category.

### Guidelines Perspective: European

Patients treated for ACS or patients of CAD with AF requiring PCI with stenting normally require DAPT with the addition of warfarin or a NOAC for primary prevention because of increased risk of stroke. The 2016 European Society of Cardiology (ESC) guidelines on AF management recommended TT of varying durations ranging from 1 month to 6 months based on the type of PCI, that is, ACS or elective and the bleeding risk. 11 This should be followed by DT. Because none of the RCTs were published till then, the guideline was not categorical on the use of NOAC in this Scenario.

However, the 2018 EHRA (European Heart Rhythm Association) practical guide on use of NOAC's give preference to NOACS over VKA as anticoagulants of choice based on evidence from RE-DUAL PCI and PIONEER-AF PCI trials. 51 The guide recommend use of TT for at least 1 month followed by DT for 1 year after stenting in stable CAD. In case of ACS, the TT can be continued beyond 1 month up to 6 months weighing the risk of stent thrombosis over bleeding risk. Both the guidelines suggest the use of clopidogrel over prasugrel or ticagrelor as bleeding risk is lower with clopidogrel based on WOEST trial. After 1 year of DT, only anticoagulants therapy is required.

The recent 2020 ESC Guidelines on AF published have deliberated on the issue of PCI in patients on OAC for chronic AF. 53 The guidelines advocate the use of NOAC in preference to VKA in conjunction with antiplatelet therapy. HAS-BLED score is again at the center stage. In patients, with high HAS-BLED score, lower NOAC doses have been advocated (15-mg rivaroxaban and 110-mg dabigatran) to curtail bleeding risk. When using VKA in, an INR target of 2.0 to 2.5 is advisable while aiming at a TTR of >70%. In patients, with low ischemic risk (stable angina or ACS undergoing uncomplicated PCI), the duration of TT is to be kept short (<1 week). For those at high risk of ischemic complications (stent thrombosis), the duration of TT can be extended up to 1 month. The guidelines also emphasize on the use of clopidogrel in combination of OAC as discussed in the section of “concomitant strategies to reduce bleeding.”

### Guidelines Perspective: American

The 2019 update of American College of Cardiology/American Heart Association/Heart Rhythm Society (ACC/AHA/HRS) guidelines on management of patients with AF in such patients recommends DT as an acceptable alternative for patients of AF who have undergone PCI (class-IIa recommendation, level of evidence B). Based on contemporary RCT's, both warfarin and NOAC's (dabigatran and rivaroxaban) are acceptable choices. 54 When TT is contemplated, it should be minimized to a duration of 4 to 6 weeks, as this is the period of greatest risk for stent thrombosis, especially in patients with ACS (class IIa, level B). They further recommend use of clopidogrel as P2Y12 for DT or TT instead of prasugrel. This is the first major guideline, the first to place DT at par with TT after acknowledging the reduced bleeding with DT. These recommendation were made before AUGUSTUS-PCI and ENTRUST AF PCI trials made their way into the literature. The positive results of these two studies only boost the case for DT.

The 2018 update of North American consensus statement on management of antithrombotic therapy in patients with AF treated with OAC undergoing PCI also clearly recommend DT as the standard regimen at discharge in all patients. 55 Additionally, NOAC's are preferred over warfarin as the oral anticoagulant of choice. TT should be reserved for selected cases with high ischemic burden and low risk for bleeding.

## Future Perspective

Two trials are undergoing to further clarify the issue of combination therapy in the scenario of AF with PCI or ACS. COACH AF PCI is an RCT of dabigatran 110-mg–based TT with VKA-based therapy (NCT03536611). The trial expects to enroll 1,120 patient and the follow-up period is 24 months.WOEST-2 registry will compare all combinations of OAC/NOAC plus all available P2Y12 inhibitors with or without aspirin therapy (NCT02635230). OPTIMA series will be a combination of two multicenter RCT's which is posed to enroll 3,742 patients (NCT03234114). OPTIMA-1 ( *n* = 2,272) will compare two TT regimens 1 month versus 6 months of durations, respectively. While OPTIMA-2 ( *n* = 1,470) will compare two dabigatran-based DT regimens with clopidogrel and ticagrelor, respectively.

## Conclusion

Approximately 15% of patients with AF have CAD and a similar amount of people with AF have coronary revascularization procedures during lifetime. Such a situation demands combination of antiplatelet and anticoagulant therapy. The use of conventional TT (warfarin + DAPT) results in reduction in both stent thrombosis and ischemic strokes alike. However, the combination results in two to three-fold increase in bleeding. Post-PCI bleeding in turn increases the odds of death by two to three times also. The use of NOAC's has resulted in tremendous reduction of bleeding events with preserved efficacy. Hence, the current scenario DT or TT with NOAC represents a potential avenue to conventional TT. Four large RCT's and three meta-analyses based on them have convincingly depicted the safety and feasibility of novel DT in the scenario of AF with PCI. Although, a small signal regarding stent thrombosis was seen, especially with a particular NOAC, the overall data for mortality and CV death was not statistically different. Hence, whenever feasible, DT based on NOAC with ultrashort TT in the initial period should be the norm. Current guidelines also place DT at par with TT considering the low bleeding potential with it. Use of lower doses of aspirin, radial access, PPI use, avoiding more potent P2Y12 inhibitor, short DAPT component, and judicious use of bleeding scores are additional measures to improve the safety of combination therapy. The duration of DT, the type of NOAC to be used and the choice of P2Y12 agent are still areas of debate and need further research.
